# Increased Risk of Thyroid Dysfunction Among Patients With Rheumatoid Arthritis

**DOI:** 10.3389/fendo.2018.00799

**Published:** 2019-01-11

**Authors:** Qian Li, Bin Wang, Kaida Mu, Jing Zhang, Yanping Yang, Wei Yao, Jie Zhu, Jin-an Zhang

**Affiliations:** ^1^Department of Endocrinology, Jinshan Hospital of Fudan University, Shanghai, China; ^2^Department of Endocrinology & Rheumatology, Affiliated Zhoupu Hospital, Shanghai University of Medicine and Health Sciences, Shanghai, China

**Keywords:** rheumatoid arthritis, thyroid dysfunction, meta-analysis, case-control study, hypothyroidism, hyperthyroidism, subclinical thyroid dysfunction, risk factors

## Abstract

**Background:** Thyroid dysfunction seems to be common among rheumatoid arthritis (RA) patients, but the risk of thyroid dysfunction in RA has not been well-defined.

**Methods:** We performed a case-control study of 65 RA patients and 550 matched non-RA subjects to assess the risk of thyroid dysfunction among Chinese RA patients. A systematic review and meta-analysis was also conducted to comprehensively define the relationship between RA and thyroid dysfunction.

**Results:** The case-control study indicated that the prevalence of thyroid dysfunction was significantly higher in RA patients than controls (OR = 2.89, *P* < 0.001). Further subgroup analyses revealed positive correlations of RA with hypothyroidism (OR = 2.28, *P* = 0.006) and hyperthyroidism (OR = 8.95, *P* < 0.001). Multivariate logistic regression analysis revealed an independent association between RA and thyroid dysfunction (Adjusted OR = 2.89, 95%CI 1.63–5.12, *P* < 0.001). Meta-analysis of 15 independent studies also showed an obviously increased risk of thyroid dysfunction among RA patients (RR = 2.86, 95%CI 1.78–4.58, *P* < 0.001). Further subgroup analysis showed RA could obviously increase risk of hyperthyroidism (RR = 2.73, 95%CI 1.29–5.77, *P* = 0.043) and hypothyroidism (RR = 2.02, 95%CI 1.49–2.74, *P* < 0.001).

**Conclusion:** Our study provides strong evidence for the increased risk of thyroid dysfunction among RA patients. Screening of thyroid dysfunction may be recommended for RA patients.

## Introduction

Rheumatoid arthritis (RA) is a chronic and systemic autoimmune disease which can result in constant inflammatory polyarthritis and continuous joint destruction, leading to damaged mobility and increased disability (1). The prevalence of RA in general population is ~1%, and RA is found to be related to some co-morbidities (2–4). The etiology of RA is still unclear and remain to be clarified, and both genetic and environment factors contribute to its etiology (5). Despite the clinical implications of RA itself, patients with RA are at increased risk of co-morbidities, for instance, cardiovascular disease (CVD) (6, 7). The mechanism underlying the increased risk of co-morbidities among RA patients is unclear, but researchers tend to attribute it to the inflammatory condition in RA patients (8, 9).

Thyroid dysfunction mainly includes hyperthyroidism and hypothyroidism. Both hyperthyroidism and hypothyroidism can be categorized into overt and subclinical stages (10–12). Hyperthyroidism is defined by excess of thyroid hormones, and Graves' disease (GD) is its main etiology. Hypothyroidism is defined by insufficiency of thyroid hormones, and its most common etiology is Hashimoto's thyroiditis (HT). Both hyperthyroidism and hypothyroidism have obviously adverse impact on human health and can lead to higher risk of cardiovascular diseases and mortality. Previous studies observed that thyroid dysfunction was prevalent in RA patients, with the prevalence ranging from 6 to 34% (13, 14). Thyroid function test is usually recommended for subjects with obvious clinical implications, like cold intolerance, loss of weight, elevated metabolism, or thyroid goiter (10, 15). Additionally, some guidelines suggest to conduct thyroid-related assessment in patients with type 1 diabetes or Addison's disease since those patients are at increased risk of thyroid dysfunction (16). However, conventional thyroid function test is not recommended in RA patients. Currently, the risk of thyroid dysfunction among RA patients has not been fully established. Herein we conducted a case-control study to evaluate both the prevalence and the risk of thyroid dysfunction among RA patients. A systematic review and meta-analysis was also conducted to comprehensively define the relationship between RA and thyroid dysfunction.

## Methods

### Participants

A total of 65 consecutive RA patients near a 3-year period were recruited from the Outpatient and Inpatient departments of Jinshan Hospital, Shanghai, China. All patients were evaluated according to American Rheumatology Association classification criteria (17). The exclusion criteria included the following: individuals with a history of other rheumatic diseases [e.g., systemic sclerosis (SS), systemic lupus erythematosus (SLE)]; active infection; malignancy and intake of drugs known to cause thyroid dysfunction. Patients who underwent thyroid surgery in the past were also excluded from the study. Furthermore, a complete assessment with emphasis on symptoms and laboratory markers of thyroid dysfunction was conducted. Written informed consent was taken from all patients in accordance with the Declaration of Helsinki. A total of 550 ethnically and geographically matched subjects were recruited from a cross-sectional study in the general population of our area, which was conducted in 2016. All of those participants were from the Chinese Han population. Either 5 or 10 non-RA controls were matched to one RA patients by age and sex. The characteristics of the subjects in this study were presented in Table 1. The protocol was approved by the Ethics Committee of Jinshan Hospital of Fudan University.

**Table 1:** Characteristic of subjects in the study.

**Variable**	**RA (*n* = 65)**	**Control (*n* = 550)**	***P***
Sex (Female, %)	52 (80.0%)	425 (77.3%)	0.62
Age (mean ± SD)^*^	59.58 ± 11.64	59.32 ± 10.72	0.86
Hypertension	23 (35.4%)	268 (48.7%)	0.04
Type 2 diabetes	4 (6.2%)	86 (15.6%)	0.05
RA duration	7.12 ± 8.58	–	–
**TREATMENT OF RA PATIENTS**			
Methotrexate	17 (26.2%)	–	–
Glucocorticoid	23 (35.4%)	–	–
Leflunomide	10 (15.4%)	–	–
Tripterygium glycosides	5 (7.7%)	–	–
NSAIDs	9 (13.8%)	–	–
Traditional chinese medicine	3 (4.6%)	–	–

* *Data was represented as mean ± SD; RA, rheumatoid arthritis; NSAIDs, non-steroid anti-inflammatory drugs*.

### Data Collection

All clinical and demographic data including sex, age, disease duration, treatment methods, C-reactive protein (CRP), rheumatoid factor (RF), anti-cyclic citrullinated peptide antibody (anti-CCP), and other variables were collected. Data of co-morbidities such as hypertension and type 2 diabetes mellitus (T2DM) were also collected. Furthermore, the level of free triiodothyronine (FT3), free thyroxine (FT4), circulating thyroid stimulating hormone (TSH), TSH receptor antibody (TRAb), antithyroglobulin antibody (TgAb), and antithyroid peroxidase antibody (TPOAb) as well as ultrasound examination and/or diffuse goiter were recorded. Thyroid dysfunction was determined by the combination of thyroid hormones and clinical symptoms, and was classified into hyperthyroidism (clinical or subclinical) and hypothyroidism (clinical or subclinical). Overt hyperthyroidism was characterized by a decreased TSH level together with elevated level of FT4, and subclinical hyperthyroidism was defined as decreased TSH level with normal FT4 level. Overt hypothyroidism was defined as an increased level of TSH and decreased FT4, while subclinical hypothyroidism (SCH) was defined as those with increased TSH level with normal FT4 level. The reference values for TSH, FT, and FT4 were 0.27–4.2 mIU/L, 3.1–6.8 pmol/L, and 12.0–22.0 pmol/L, respectively.

### Statistical Analysis

All data were analyzed by Stata (version 12.0, StataCorp). The difference in sex, hypertension and T2DM between RA patients and controls were assessed using Chi-square test, and the difference in age was determined using *t*-test. The frequency of thyroid dysfunction between RA patients and controls was compared with Chi-square test, and odds ratio (OR) with 95% confidence interval (95%CI) was calculated. Multiple logistic regression analysis was also conducted to evaluate the association between thyroid dysfunction and RA. Because both hypertension and T2DM were prevalent among RA patients and controls and there was some difference in their proportions, we thus conducted logistic regression analysis with adjustment for age, gender, hypertension and T2DM. Moreover, logistic regression analysis was also performed to assess the impact of disease duration on risk of thyroid dysfunction among RA patients. A *P* < 0.05 was considered statistically significant.

### Systematic Review

The systematic review and meta-analysis were done in accordance with the PRISMA guideline (18). Pubmed and Embase were searched with the following search strategy: (rheumatoid arthritis) AND (thyroid dysfunction OR hypothyroidism OR hyperthyroidism). There was no language restriction. The reference lists of included studies were also searched. We included observational studies, such as cohort studies, cross-sectional studies, and case-control studies, which compared the prevalence of thyroid dysfunction between RA patients and non-RA controls. Eligible studies must include RA patients and non-RA controls, while those enrolled patients with juvenile arthritis or psoriatic arthritis were excluded. The outcome of interest was the relative risk of thyroid dysfunction among RA patients compared with non-RA controls. Included studies need to provide risk estimates with 95%CI for the association between RA and thyroid dysfunction, such as relative risk (RR) and OR, or provide other data which could be transformed into risk estimates. Case reports and studies containing overlapping data were excluded.

Data extraction was conducted using an extraction form, which mainly included study characteristics, participant characteristics, types of thyroid dysfunction and adjusted cofounders or matched factors. Quality assessment of included studies was conducted using Newcastle-Ottawa scale (NOS), and was based on participant selection, exposure evaluation, outcome evaluation and cofounders adjustment (19). Studies scoring 5 or fewer points were defined as studies with suboptimal quality, and those with 6 or more points had good quality. RR with 95%CI was used to evaluate the risk of thyroid dysfunction among RA patients. Heterogeneity was assessed using the I2 statistic, and I2 > 50% was deemed as high heterogeneity. Data were pooled using random-effects meta-analysis (20). Subgroup analysis was conducted based on type of hypothyroidism (Overt hypothyroidism; SCH). Publication bias was assessed by the funnel plot and Egger's test. Trim and fill method was utilized when publication bias existed. All analyses were conducted in Stata (version 12.0, StataCorp), and *P* < 0.05 was considered statistically significant.

## Results

### Case-Control Study

Among the RA patients enrolled in the study, 80.0% were female, and the mean age was 59.58 years. Both hypertension and T2DM were prevalent among RA patients and controls, and there was some difference in their proportions (Table 1). The profile of distinct subsets of thyroid dysfunction was displayed in Table 2. Among the RA patients, 21 (32.3%) were diagnosed with thyroid dysfunction, 4 (6.2%) of which were hyperthyroidism and the others were hypothyroidism (26.2%). In contrast, only 14.2% individuals in the control group (78 cases) had thyroid dysfunction, including 4 (0.7%) hyperthyroidism cases and 74 (13.5%) hypothyroidism cases. Compared with the control group, the percentage of thyroid dysfunction was significantly increased in RA group (OR = 2.89, 95%CI 1.63–5.12, *P* < 0.001), which illustrated the increased risk of thyroid dysfunction in RA patients. Additionally, the proportions of hyperthyroidism and hypothyroidism in RA group were also increased compared with that in the control group (OR = 8.95, *P* < 0.001; OR = 2.28, *P* = 0.006). Furthermore, in comparison with controls, the number of subjects with overt hypothyroidism was significantly increased in RA group (*P* < 0.001) (Table 2).

**Table 2:** The prevalence of thyroid dysfunction and its subgroup in RA patients and controls.

	**RA group (*n* = 65)**	**Control group (*n* = 550)**	***P***	**OR**	**95%CI**
Thyroid dysfunction	21 (32.3%)	78 (14.2%)	< 0.001	2.89	1.63–5.12
Hyperthyroidism	4 (6.2%)	4 (0.7%)	< 0.001	8.95	2.18–36.70
Overt hyperthyroidism	3 (4.6%)	4 (0.7%)	0.005	6.61	1.45–30.19
Hypothyroidism	17 (26.2%)	74 (13.5%)	0.006	2.28	1.24–4.17
Overt hypothyroidism	7 (10.8%)	4 (0.7%)	< 0.001	16.47	4.68–57.96
Subclinical thyroid dysfunction	11 (16.9%)	70 (12.7%)	0.344	1.40	0.70–2.80
Subclinical hyperthyroidism	1(1.5%)	0 (0%)	0.05	25.60	1.03–635.1
Subclinical hypothyroidism	10 (15.4%)	70 (12.7%)	0.547	1.25	0.612.56

*RA, rheumatoid arthritis*.

Outcomes from logistic regression analysis were shown in Table 3. In general, the results indicated that RA was independently associated with thyroid dysfunction (OR = 3.03, 95%CI 1.68–5.48; *P* < 0.001). Further subgroup analysis showed that RA patients were at greater risk of hypothyroidism (OR = 2.35, 95%CI 1.26–4.40; *P* = 0.007), especially for overt hypothyroidism (OR = 17.95, 95%CI 4.88–66.01; *P* < 0.001). Moreover, RA was also independently associated with increased risk of hyperthyroidism (OR = 11.30, 95%CI 2.54–50.29, *P* = 0.001) and overt hyperthyroidism (OR = 7.75, 95%CI 1.56–38.59; *P* = 0.012). Logistic regression analysis in RA patients revealed that disease duration had no obvious impact on the risk of thyroid dysfunction (Adjusted OR = 0.95, 95%CI 0.88–1.02; *P* = 0.17).

**Table 3:** Outcomes in the relationship between RA and thyroid dysfunction from the logistic regression analysis.

	***P***	OR^*^	**95%CI**
Thyroid dysfunction	< 0.001	3.03	1.68–5.48
Hyperthyroidism	0.001	11.30	2.54–50.29
Overt Hyperthyroidism	0.012	7.75	1.56–38.59
Hypothyroidism	0.007	2.35	1.26–4.40
Overt Hypothyroidism	< 0.001	17.95	4.88–66.01
Subclinical thyroid dysfunction^#^	0.340	1.41	0.69–2.89
Subclinical Hypothyroidism	0.555	1.25	0.60–2.61

* *Adjusted factors in the logistic regression analysis included age, gender and hypertension*.

# *Subclinical thyroid dysfunction included subclinical hyperthyroidism and subclinical hypothyroidism*.

### Meta-Analysis

**Figure 1:**
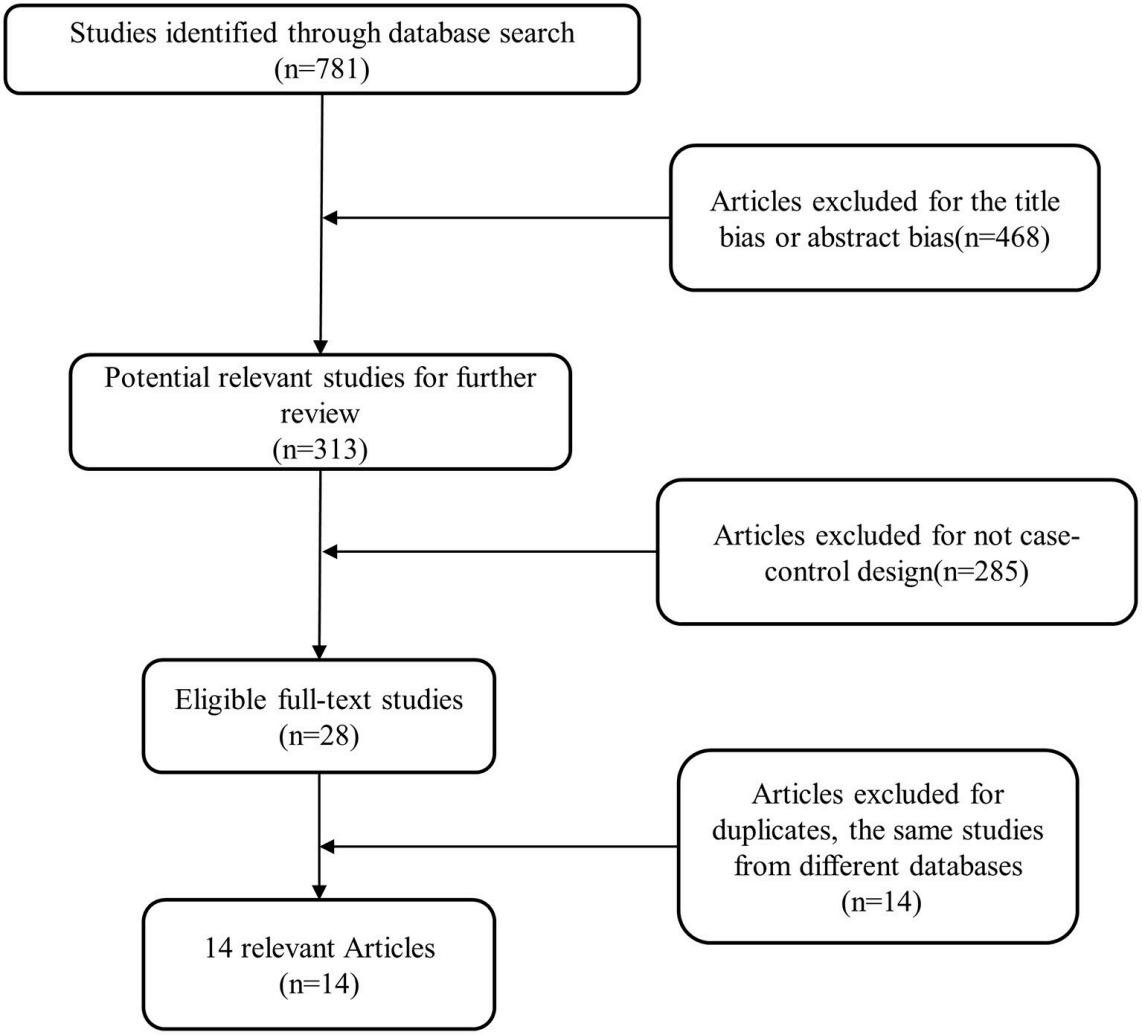
The detailed procedures of study selection in the meta-analysis.

A total of 15 relevant reported studies together with our study were included in the meta-analysis. The detailed steps of selecting research was depicted in Figure 1. According to the selection criteria, 1,017 studies were excluded and 15 studies were included in the present study.

**Figure 2:**
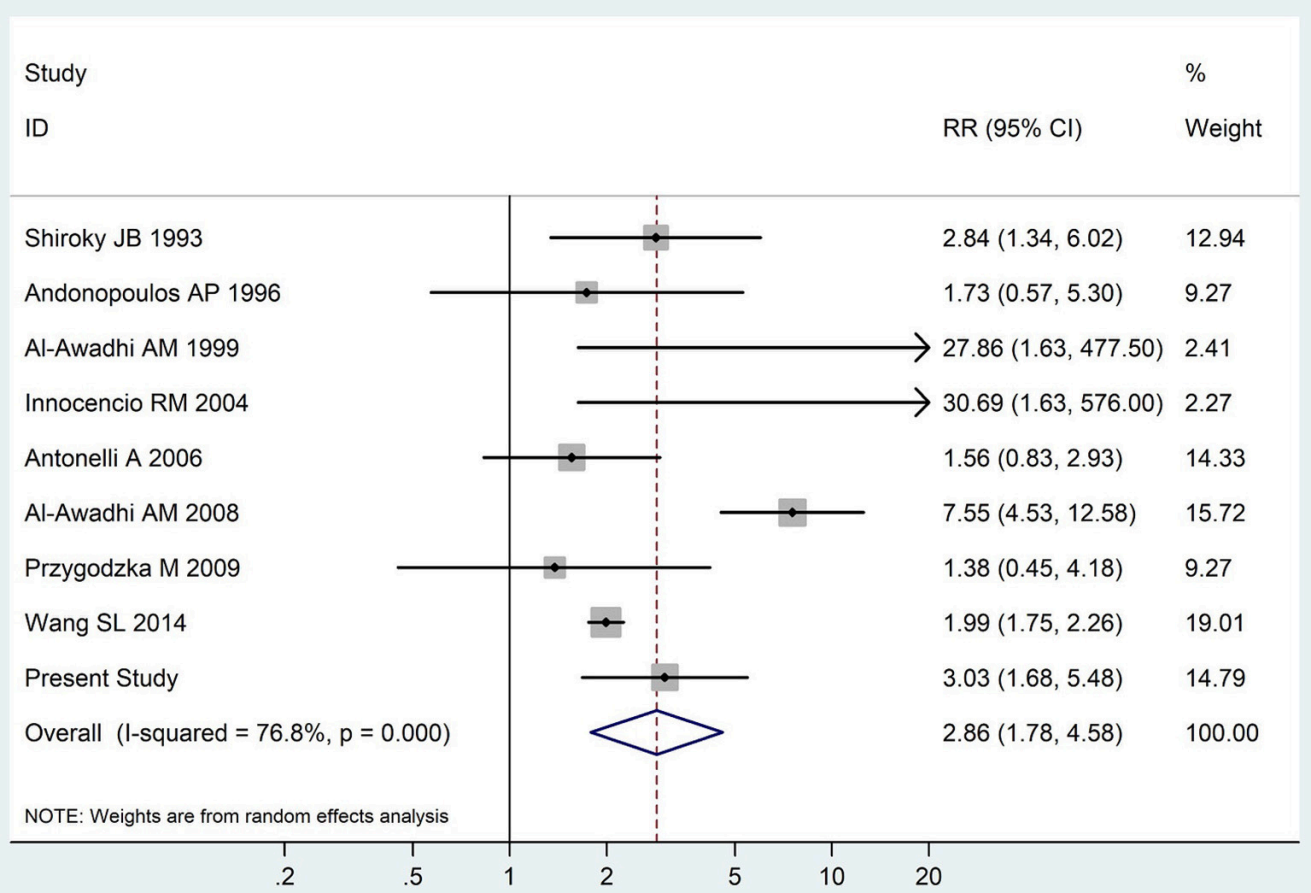
Forest plot for the increased risk of thyroid dysfunction among RA patients (The diamond represents the pooled RR and 95% CI).

**Figure 3:**
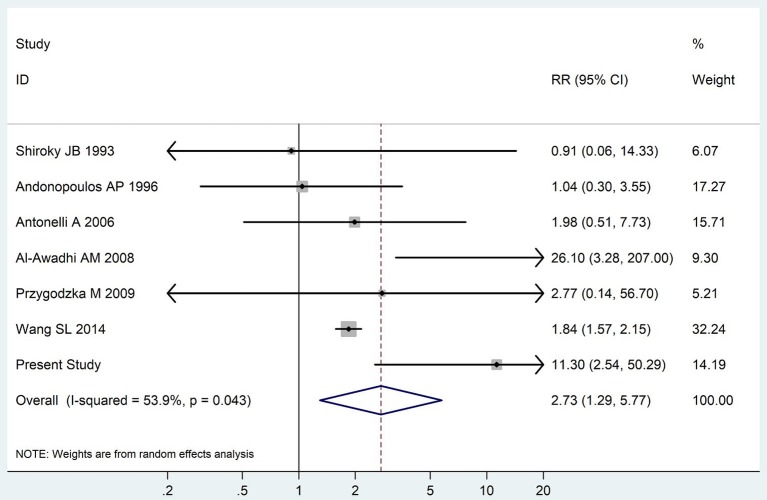
Forest plot for the increased risk of hyperthyroidism among RA patients (The diamond represents the pooled RR and 95% CI).

**Figure 4:**
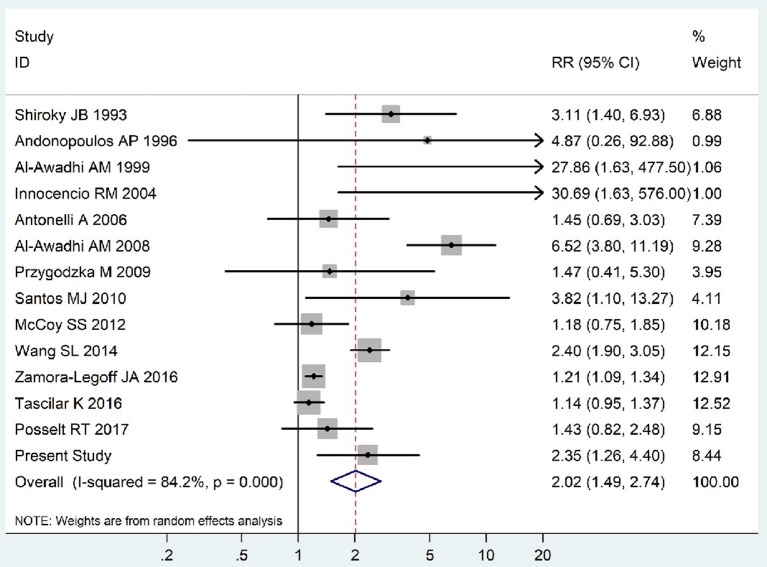
Forest plot for the increased risk of hypothyroidism among RA patients (The diamond represents the pooled RR and 95% CI).

**Figure 5:**
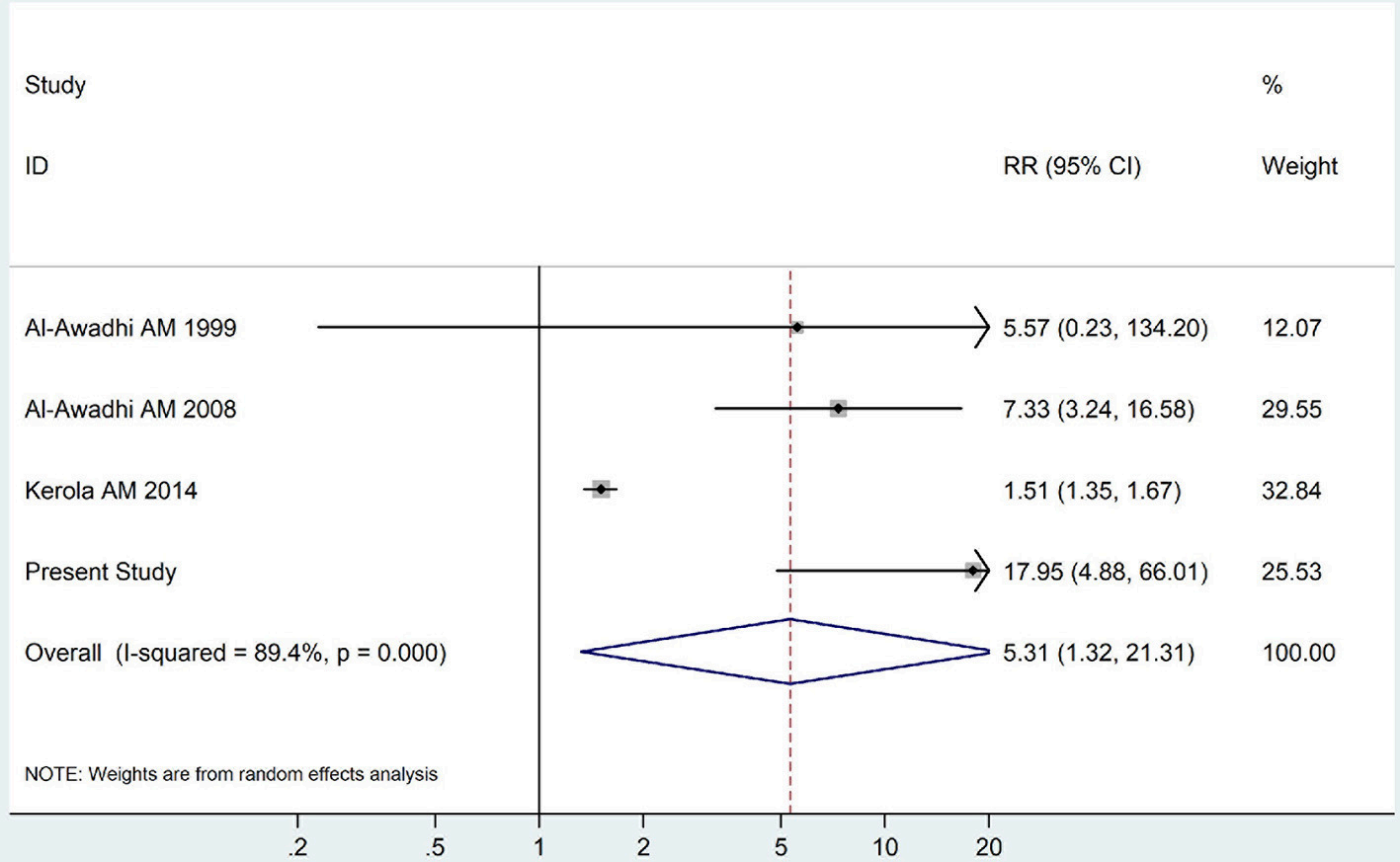
Forest plot for the increased risk of overt hypothyroidism among RA patients (The diamond represents the pooled RR and 95% CI).

**Figure 6:**
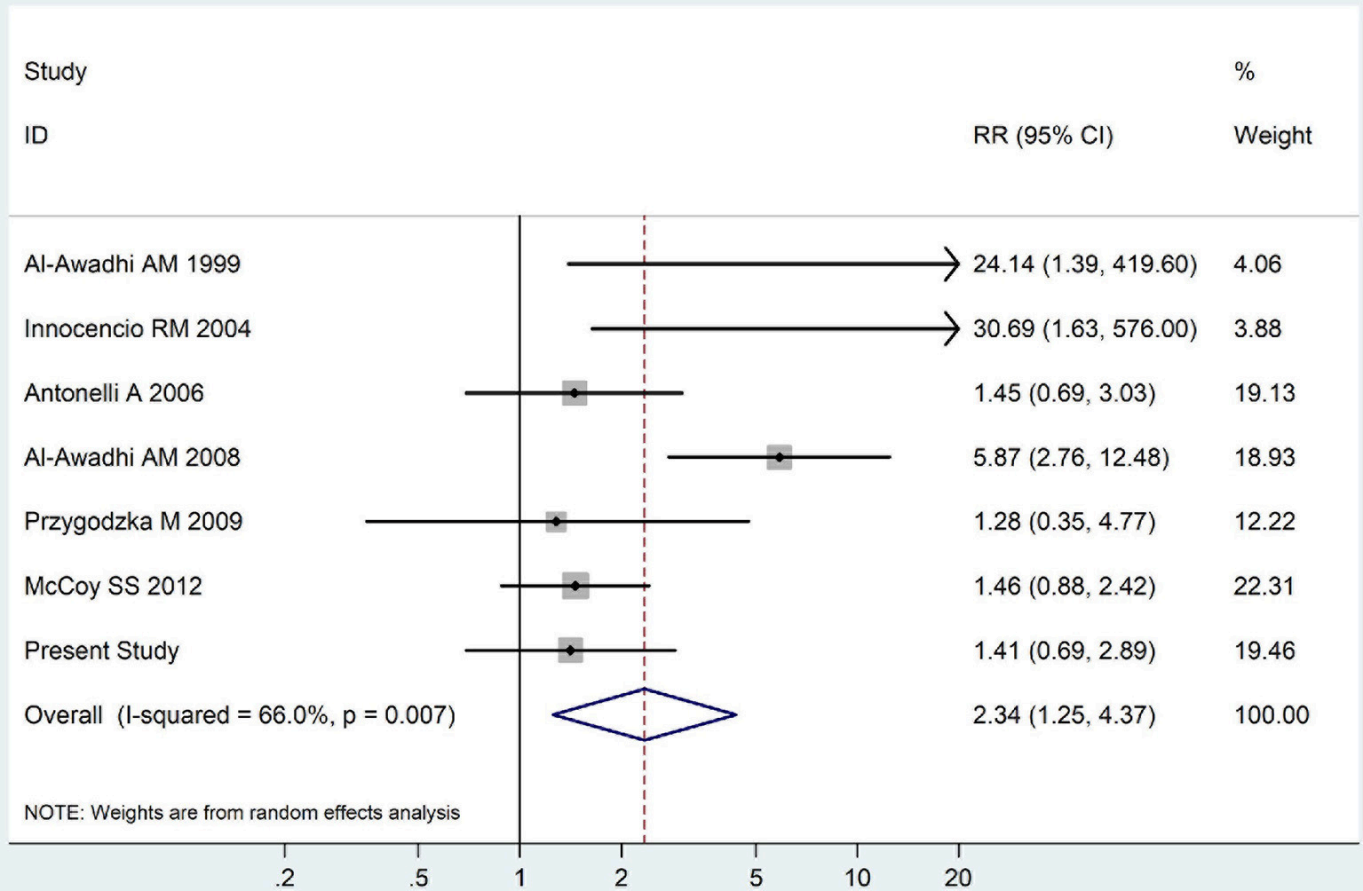
Forest plot for the increased prevalence of subclinical thyroid dysfunction among RA patients (The diamond represents the pooled RR and 95% CI).

The summary of those 15 studies was displayed in Table 4. The meta-analysis demonstrated an significantly increased risk of thyroid dysfunction among RA patients (RR = 2.86, 95%CI 1.78–4.58; *P* < 0.001; Figure 2). Further subgroup analysis revealed RA could obviously increase risk of hyperthyroidism (OR = 2.73, 95%CI 1.29–5.77; *P* = 0.043; Figure 3), hypothyroidism (OR = 2.02, 95%CI 1.49–2.74; *P* < 0.001; Figure 4). Furthermore, the meta-analysis also indicated a strong correlation between overt hypothyroidism and RA, and the pooled RR was 5.31 (95%CI 1.32–21.31; *P* < 0.001; Figure 5). In addition, the analysis proved that RA patients were likely to have increased prevalence of subclinical thyroid dysfunction which including subclinical hyperthyroidism and clinical hypothyroidism, and the RR was 2.34 (95%CI 1.25–4.37; *P* = 0.007; Figure 6).

**Table 4:** Characteristics of 15 studies included in the meta-analysis.

**Author**	**Year**	**Region**	**Participants**		**Types of thyroid dysfunction**	**Study design**
			**RA**	**Control**		
Shiroky JB	1993	Canada	119	108	Dysfunction Hypothyroidism Hyperthyroidism	Case-control
Andonopoulos AP	1996	Greece	101	70	Dysfunction Hypothyroidism Hyperthyroidism	Case-control
Al-Awadhi AM	1999	Kuwait	48	90	Dysfunction Hypothyroidism SCH Overt hypothyroidism	Case-control
Innocencio RM	2004	Brazil	25	113	Dysfunction Hypothyroidism SCH	Case-control
Antonelli A	2006	Italy	91	180	Dysfunction Hypothyroidism SCH Hyperthyroidism	Case-control
Al-Awadhi AM	2008	Kuwait	177	577	Dysfunction Hypothyroidism SCH Overt hypothyroidism Hyperthyroidism	Case-control
Przygodzka M	2009	Poland	100	55	Dysfunction Hypothyroidism SCH Hyperthyroidism	Case-control
Santos MJ	2010	Portugal	98	102	Hypothyroidism	Case-control
McCoy SS	2012	USA	650	650	Hypothyroidism SCH	Cohort
Kerola AM	2014	Finland	7209	None	Overt hypothyroidism	Case-control
Wang SL	2014	Taiwan	3657	14628	Dysfunction Hypothyroidism Hyperthyroidism	Case-control
Zamora-Legoff JA	2016	USA	497	527	Hypothyroidism	Case-control
Tascilar K	2016	Canada	1357	13570	Hypothyroidism	Case-control
Posselt RT	2017	Brazil	210	141	Hypothyroidism	Case-control
Present Study	2017	China	65	550	Dysfunction Hypothyroidism Hyperthyroidism Overt hypothyroidism SCH	Case-control

## Discussion

RA is a systemic and chronic disease which leads to symmetrical polyarthritis and progressive destruction of joint. The disease leads to disability and mortality if not treated. In our case-control study, we investigated 65 RA patients and 550 matched healthy individuals to evaluate the relationship between RA and thyroid dysfunction. Herein we found that patients with RA were likely to have increased prevalence of both hyperthyroidism and hypothyroidism, especially overt hypothyroidism. Subsequently, we performed a meta-analysis containing 15 distinct studies to further explore the relationship between RA and thyroid dysfunction, and found that RA was an important risk factor of thyroid dysfunction.

Thyroid dysfunction includes hypothyroidism and hyperthyroidism and is mainly induced by autoimmune thyroid disease (AITD) such as GD or HT (21, 22). Intriguingly, thyroid dysfunction is frequently accompanied by non-thyroid autoimmune diseases (22). The relationship between RA and thyroid dysfunction has been studied extensively in decades, but there is still lack of a definite conclusion (23). Numerous studies had looked into the correlation between RA and thyroid dysfunction and the earliest research can be traced back to 1,963 (24). By investigating 101 RA and 70 controls, Andonopoulos et al. (25) found that the level of thyroid autoantibodies in RA patients was different from the controls. However, it failed to find significant difference between thyroid dysfunction and RA. Recently, a study in 2014 evaluated the association between rheumatic diseases and thyroid disorders, which suggested that thyroid-related antibodies were significantly increased in RA patients as well the occurrence of thyroid dysfunction (26). In our case-control study, we found that the prevalence of thyroid dysfunction in RA patients differed significantly from the controls, and RA patients had an increased prevalence of hypothyroidism. This result was in line with a recent study published in 2017, which demonstrated an obvious correlation between hypothyroidism and RA (27). In previous studies, more outcomes revealed the correlation between RA and hypothyroidism, but not hyperthyroidism. Our case-control study found that the incidence of hyperthyroidism in RA patients was significantly different from the controls, which provided evidence that RA patients may have the disposition toward hyperthyroidism. Except for RA, some researchers had revealed some correlations of thyroid disorders with other autoimmune diseases like SLE, SS and multiple sclerosis (MS) (28, 29). One previous research revealed the correlation between SLE and thyroid diseases (28). In 2010 (29), Alessandro and his colleagues found a higher occurrence of overt hypothyroidism and GD in female SLE patients. Recently, Liu et al. (30) observed a higher prevalence of thyroid disease in SLE patients compared to healthy controls via a large cohort study. Likewise, numerous studies found high prevalence of thyroid disorders among SS or MS patients (31–34).

In order to examine the association between RA and thyroid dysfunction statistically and quantitatively, we performed a meta-analysis consisting of 15 independent relevant studies, which revealed a strong correlation between RA and elevated risk of thyroid dysfunction. The meta-analysis also demonstrated a rather positive correlation between RA and elevated risk of overt hypothyroidism, which indicated the clinical importance of conducting thyroid function tests in RA patients. Distinct with the results from our case-control research, the meta-analysis showed a positive relationship between RA and SCH, and a possible reason may be the limited sample size of our study.

The exact mechanism underlying the relationship between RA and thyroid disorders is still unclear. As autoimmune diseases, the etiology of RA and AITD are complex, both genetic and environmental factors are involved (35–39). In line with this, autoimmune diseases generally share similar pathological pathways, which implies the possible aggregation phenomenon of autoimmune diseases. Some gene variations were found to exert great effect in the pathogenesis of both RA and AITD. For instance, numerous studies revealed that single nucleotide polymorphisms (SNPs) of *HLA-DRB1* (40–46), *STAT4* (47–52), and vitamin D receptor (*VDR*) (53–58) were associated with RA and AITD. Scientists also observed that smoking and vitamin D deficiency had strong association with RA and AITD (59–61). Furthermore, some researchers found that similar immune dysregulation appears in both RA and AITD. Our earlier study demonstrated the imbalance between T helper cell 17 (Th17) and regular T cells (Treg) led to AITD (62), and other studies revealed similar pathogenic role of Th17/Treg imbalance in RA (63, 64). Therefore, because thyroid dysfunction is mainly resulted from AITD, the increased risk of thyroid dysfunction in RA patients may be explained by those shared factors involved in the pathogeneses of RA and AITD.

Several limitations of this study need to be taken into consideration. First, the sample size of the case-control study was relatively small, which may lead to deficient statistical power in subgroup analysis. Second, we conducted thyroid functional tests at the recruitment time and did not perform an investigation to monitor thyroid function during follow-up. Since there is a possible assumption that the RA duration may increase the disposition toward thyroid dysfunction, the risk of thyroid dysfunction may differ in RA patient with various durations, which need to be elucidated in future studies. Finally, the impact of thyroid dysfunction on RA prognosis or treatment outcomes is still not well-established. The finding from logistic regression analysis in RA patients revealed that disease duration had no obvious impact on the risk of thyroid dysfunction, which was not reliable because of the limited number of RA patients. Therefore, future studies recruiting more RA patients are necessary to determine the impact of disease duration on risk of thyroid dysfunction.

As one of the autoimmune and inflammatory disease, glucocorticoid was widely used to alleviate the inflammation and suppress over-active immune responses in RA patients (65–67). In general conditions, the usage of glucocorticoid exerts litter influence on the regulation of thyroid hormone. However, under some circumstances, the utilization of high-dose glucocorticoid leads to a direct suppression of TSH secretion without increasing FT3 and FT4 (68). Leflunomide, as another drug for the treatment of RA, could affect thyroid function (69). The above data suggest that intake of some drugs for RA may also increase risk of thyroid dysfunction. However, current observational studies which aimed to assess the impact of those drugs on thyroid dysfunction among RA patients were relatively small, further studies with larger sample size are warranted.

In summary, our study reveals an increased prevalence of thyroid dysfunction in RA patients, and RA is an important risk factor for thyroid dysfunction. RA patients are at a higher risk of thyroid dysfunction, especially overt hypothyroidism. Hence, this study suggests that thyroid-related examinations including thyroid auto-antibodies and thyroid function tests should better be included when evaluating RA patients, and further articles to elucidate the mechanisms underlying the relationship between RA and thyroid dysfunction are necessary.

## Ethics Statement

The research project was approved by the Ethics Committee of the Jinshan hospital, the Institutional Review Board of Jinshan Hospital of Fudan University (No. 2015-KY12-457).

## Author Contributions

QL wrote the article. JinZ supervised the whole experiments and the writing of the article. BW helped to designed the experiment. KM, JingZ, YY, WY, and JieZ helped to analyses the data.

### Conflict of Interest Statement

The authors declare that the research was conducted in the absence of any commercial or financial relationships that could be construed as a potential conflict of interest.
